# Vector Potential of* Blattella germanica* (L.) (Dictyoptera: Blattidae) for Medically Important Bacteria at Food Handling Establishments in Jimma Town, Southwest Ethiopia

**DOI:** 10.1155/2016/3490906

**Published:** 2016-05-11

**Authors:** Fithamlak Solomon, Fanuel Belayneh, Gebre Kibru, Solomon Ali

**Affiliations:** ^1^School of Medicine, Wolaita Sodo University, Sodo, Ethiopia; ^2^School of Public Health, Wolaita Sodo University, Sodo, Ethiopia; ^3^Department of Medical Laboratory Sciences and Pathology, Public Health and Medical Science College, Jimma University, Jimma, Ethiopia

## Abstract

Cockroaches have been regarded as possible vectors of human enteropathogens. Their presence and crawl particularly in food handling establishments could be risky for human health. Therefore, this study was done to determine the vector potential of cockroach for medically important bacterial pathogens in restaurants and cafeterias. A cross-sectional study was conducted on cockroaches from restaurants and cafeterias in Jimma town from May to September 2014. Standard taxonomic keys and microbiological techniques were applied for species identification and isolation. Data was analyzed in SPSS version 16.0. All cockroaches trapped were the German cockroach,* Blattella germanica* (L.) (Dictyoptera: Blattidae).* Escherichia coli* was the most frequently isolated followed by* Salmonella* species (serogroups B, D, E, C_1_, and NG),* Bacillus cereus*, and* Shigella flexneri*. Wide varieties of bacteria of medical relevance were also identified. Of which,* Klebsiella* spp. 49(40.8%),* Bacillus* spp., and* Staphylococcus saprophyticus* were predominant.* Blattella germanica* (L.) (Dictyoptera: Blattidae) could serve as a potential vector for the dissemination of foodborne pathogens such as* Salmonella* spp.,* Shigella flexneri*,* E. coli*,* S. aureus*, and* B. cereus* and these bacteria could be a major threat to public health. Therefore, environmental sanitation and standard hygiene need to be applied in the food handling establishments in that locality.

## 1. Introduction

Cockroaches have long been regarded as possible vectors of human enteropathogens owing to their unsanitary life style and their indiscriminate feeding on sanitary wastes and human meals [1].

Various investigations all around the world also revealed that cockroaches living close to human dwellings were important carriers of etiologic agents belonging to all groups of potential humans and animals pathogens including bacteria, protozoa, worms, fungi, and viruses [2]. Exposure to cockroach antigens also plays an important role in allergens and asthma related health problems [2, 3]. They are known to carry about 57 species of pathogenic bacteria, of which* Escherichia coli*,* Staphylococcus aureus*,* Shigella*, and* Salmonella* species are most common foodborne pathogens encountered. But their role in the direct transmission of infection has seldom been established [4, 5].

The range of problems caused by the presence of cockroaches varies between areas they inhabit: the fewer the bacterial species present in the environment, the fewer the bacteria cockroaches will carry [6, 7]. After feeding on contaminated food, bacteria or protozoa can remain in the cockroach external body or within the cockroaches' digestive system for a month or more and later be transmitted in their faeces [8].

Though various studies indicated that domestic cockroaches play an important role as mechanical as well as biological vectors for foodborne bacterial pathogens, there is no adequate information in the study area in particular and in the country in general. Therefore, the aim of this study was to isolate medically important bacteria from cockroaches. This study was also designed to determine the species composition of cockroaches which are found at food handling establishments (FHEs) of Jimma town.

## 2. Materials and Methods

### 2.1. Study Area

This community based cross-sectional study was done at FHEs (all restaurants and cafeterias) of Jimma town from May 1 to September 30, 2014. The town is located at 1780 m above sea level. The area is generally characterized by warm and humid climate. The mean annual maximum temperature of the town is 30°C with a mean annual minimum temperature of 14°C [9]. The annual rainfall ranges from 1138 mm to 1690 mm. Maximum precipitation occurs during the three months' period, June to August, with minimum rainfall in December and January. The total population of the town that settled on 50.52 km^2^ area was estimated to be 149,164 [10]. This gives an average population estimate per square kilometer area of 2952.5 [10]. During the study period, there were about 56 restaurants and 29 cafeterias with different levels, capacity, or standard. Many of these FHEs are located in densely populated areas where there is no adequate access for latrine, poorly constructed houses, and a reduced environmental sanitation. Cockroach infestation is high in the town.

### 2.2. Ethics

Approval and permission to study were obtained from Institutional Review Board (IRB) of College of Public Health and Medical Sciences of Jimma University. Support letter for all cafeterias and restaurants was written by the Jimma town municipality. Consent was also obtained from the owner of each of the cafeterias and restaurants. All information about the food handling establishments (FHEs) was kept confidential.

#### 2.2.1. Cockroaches Trapping

Hoy-Hoy roach sticky traps (Pecopp Pest Control Service) were used to trap cockroaches in FHEs which are ideal for monitoring levels of cockroach infestation. It has a sticky floor approximately 15 cm × 10 cm with an attractant sachet (cyclotene coated sex pheromone) to entice cockroaches to enter the trap. Because of its safety, ease of use, and nontoxicity sticky trap is considered to be a valuable tool in cockroach integrated pest management programs [11]. Sticky traps were placed in kitchens, food preparation areas, refrigerators, coffee machines, bathrooms, and toilets in the selected restaurants and cafeterias. Traps were placed in areas which were hard to reach and dark and crammed corners and tight against the wall.

During trapping of cockroach, five sticky traps were placed in different sections of the FHEs each with a minimum of five feet apart. The sticky traps were placed at 18:00 hrs and left overnight and retrieved from 7:00 to 9:00 hrs in the morning. Sticky traps containing cockroaches were picked from the FHEs with sterile surgical glove, sealed in sterile plastic bag, and placed on their side in order to avoid crushing of the captured cockroaches. They were then transported to the Jimma University microbiology laboratory by cold chain system for investigation. During five months of study period, cockroach sticky traps were placed at 49 restaurants and 27 cafeterias once a week. Accordingly, a total of 1140 adult cockroaches, 730 from restaurants and 410 from cafeterias, were included for microbiological investigation. Since the cockroach infestation is high and cockroaches may probably carry the same amount of bacteria cockroaches were taken as a pool of 10 cockroaches as one batch [8, 12–14].

#### 2.2.2. Cockroach Fauna Identification

Species of domestic cockroach was identified by using standard taxonomic keys which include size, color of the wing, and pronotum appearance of the exocuticle, tegmina, wing, and femur with comparison of color pictures of cockroaches [15].

### 2.3. Microbiological Sample Preparation

The trapped cockroaches were picked from the sticky traps by sterile forceps and pooled in batches (i.e., ten cockroaches pooled as one sample) and anesthetized in a sterile jar by using chloroform soaked cotton [13]. The immobilized cockroaches were placed in 5 mL sterile physiological saline (0.85%) and shaken for two minutes to dislodge bacteria from their body surfaces. Then, the wash was taken as external body homogenate sample and checked for bacteria growth [16]. Subsequently, those dislodged cockroaches were soaked in 90% ethanol for five minutes to decontaminate their external surfaces and dried. After that, they were rewashed with sterile physiological saline (0.85% NaCl) in order to remove traces of ethanol. Then, the cockroaches' alimentary tract was aseptically dissected out using autoclave-sterilized entomological dissecting needles under a binocular dissecting microscope. The instruments were dipped in ethanol and flamed between dissections. The excised gut was then homogenized in 5 mL sterile normal saline and the homogenates were used for bacteriological analysis [16].

#### 2.3.1. Isolation and Identification of Foodborne Bacterial Pathogens

One mL of each of the external and gut homogenate samples was suspended separately into 9 mL of sterile bottles containing buffered peptone water (Oxoid, Hampshire, UK) and a homogenous enrichment suspension was prepared in nutrient broth and incubated at 37°C for 18–24 h. The preenriched yield solutions then separately inoculated on MacConkey, xylose lysine deoxycholate (XLD) agar, mannitol salt agar (MSA), and polymyxin-B egg yolk mannitol bacillus cereus agar (PEMBA). Rappaport-Vassiliadis (RV) broth was also used as a primary enrichment medium for the identification of* Salmonella* and* Shigella*. Gram-negative bacterial rods showing growth on MacConkey agar were subcultured to nutrient agar after picking 3–5 colonies to obtain pure colonies and were grown again to the original media for colony identification by incubating at 37°C for 24 h. The bacteria growth on the agar media was identified by colonial morphology, Gram-staining, and a battery of biochemical tests (oxidase, catalase, Simmons citrate, indole production, urease, motility, coagulase, methyl red-Voges Proskauer (MR-VP), lysine decarboxylase (LDC), Klingler's iron agar (KIA), mannitol fermentation, gas, and H_2_S production) [17, 18]. Serogrouping of* Salmonella* spp. was done by slide agglutination technique using polyvalent and monovalent (O_2_, O_3_, O_4_, O_5_, O_6_, O_7_, O_8_, O_9_, O_15_, and Vi) antigens for identification of* Salmonella* serogroups, A–E (Difco, Detroit, USA). Similarly,* Shigella* serogrouping was done using known polyvalent* Shigella* antisera A, B, C, and D (Remel, Europe Ltd., UK). Physiological saline was used in the test as a negative control [19].

### 2.4. Data Analysis

The data was edited, coded, and entered into SPSS for Windows version 16.0. Descriptive statistics were used to determine frequency, prevalence, and percentage. The relationship between variables was computed using chi-square (with *P* value of 0.05 and 95% confidence interval).

## 3. Results

During the study period, a total of 1841 cockroaches (both adult and nymph) were trapped from different sections of the food handling establishments (FHEs) in Jimma town and from these 1140 adult cockroaches were included for laboratory investigation. Based on standard taxonomic keys for cockroach fauna identification criteria, all cockroaches were identified as German cockroach,* Blattella germanica* (L.) (Dictyoptera: Blattidae).

Relatively high proportion of cockroach infestation was detected in kitchens of restaurants (49.9%) and coffee machines of cafeterias (40%). Least infestation rate (4.5%) was identified in the compartment of refrigerators in both FHEs (Table 1).

Out of 114 pooled cockroaches (a pool of ten cockroaches as one sample), 114 external homogenates, and 114 internal homogenates (228 totally) investigated for bacteriology, 91 foodborne bacteria were isolated, of which 39 were from the external surfaces and 52 were from guts of cockroaches (Table 2). Among foodborne bacterial pathogens,* E. coli* was predominant followed by* Salmonella* spp. and* B. cereus* (Table 2). Of those* Salmonella* serogroups, group B was most frequently seen followed by serogroups D and E.

In this study, no statistical association was observed between isolated foodborne bacteria and type of FHEs (*P* = 0.87). Moreover, there was no significant difference between body parts (external or gut surfaces) of cockroaches and frequency of foodborne bacterial isolates (*P* = 0.172).

Apart from these, wide variety of Gram-negative and Gram-positive bacteria of medical importance were also identified from both surfaces of cockroaches. From the external surface isolates,* Klebsiella* species 49 (43%),* Enterobacter* spp. 25 (21.9%), and* coagulase negative staphylococci* 25 (21.9%) were the most frequently isolated ones. From gut bacterial harborage was found from as high as 44 (38.6%) for* Klebsiella* spp. and 41 (36%) for* Bacillus* species to as low as 6 (5.3%) for* S. marcescens* and 5 (4.4%) for* Edwardsiella tarda* (Table 3).

## 4. Discussion

The* Blattella germanica* are the most abundant and closely associated insects with humans worldwide [20]. Though there is no sufficient data on cockroach species surveillance in Ethiopia,* Blattella germanica* cockroaches were reported at Ziway and Addis Ababa districts [21]. The only active cockroaches that forage in the FHEs of Jimma town were found out to be* Blattella germanica*, which is consistent with the findings of studies at FHEs and hospitals in Addis Ababa [13, 14]. The preference of* Blattella germanica* cockroaches to inhabit in tropical and subtropical weather condition might make Jimma town suitable habitat for this species.

The isolation of higher rate (57.1%) of foodborne bacterial pathogens in the gut compared to the external surfaces in this study goes in agreement with study findings reported in Ethiopia [13], Libya [22], and Taiwan [23] where 57%, 51%, and 50.8% gut bacterial carriage were noted, respectively. However, our finding is contrary to the carriage rates reported in Nigeria where 69.7% [24] and 52.9% [25] external surface bacterial carriage were documented. The relative high external surface carriage reported in these studies in Nigeria could be due to the use of faecal pellets as internal homogenate sample where bacteria may remain in the gut and take longer time to be excreted through faeces.

In the present study, cockroaches harbored bacterial pathogens associated with foodborne illness and food spoilage organisms via their bodies. Considering this fact, cockroaches could be the potential vectors/reservoirs for foodborne bacteria pathogens.

The isolation of* Salmonella* in the present study was similar to report made in Ghana [26], India [27], Iran [28], and Addis Ababa [13]. It is known that* Salmonella* serogroups A–E are responsible for 99% of the human salmonellosis [29]. The* Salmonella* serogroups identified in this study go in agreement with findings of Tachbele et al. where* Blattella germanica* cockroaches collected at restaurants and hospitals of Addis Ababa carried* Salmonella* serogroups B, D, and E [13]. Similarly* Salmonella* C_1_ was identified from cockroaches trapped at pediatrics unit of hospitals in Addis Ababa too [14]. Since cockroaches are passive carriers of bacterial pathogens in the environment where they reside, the identification of* Salmonella* serogroups in our study might reflect their prevalence in food and FHEs.

The* S. flexneri* isolate detected from cockroaches body substantiated the previous report made in Ghana [26], Botswana [12], Iran [30], and Ethiopia [14]. The isolation of* S. flexneri* from the gut of cockroaches in the FHEs might be a reflection of recent infected source as dose feeding experiments demonstrated that maximum survival shedding duration of cockroaches for* Shigella* was less than four days after they fed with standard inoculums via contaminated food [31]. If not,* Shigella* spp. may survive longer in cockroaches' gut and could serve as a reservoir host for this bacterium eventually creating a suitable environment for survival and dissemination. Cockroaches' vector competence for carriage of* S. flexneri* is a considerable threat for a consumer and the community since cockroaches could defecate these highly pathogenic bacteria as they rest and could regurgitate them as they eat.

In this study* E. coli* were the most frequently identified bacteria associated with foodborne illness. Similar report was made in India [16], Ethiopia [13], Iran [2, 30], and Nigeria [25].

The presence of* S. aureus* on cockroaches in our study has indicated that they could play a role in the dissemination of this bacterium. This finding was analogous with a report made in Taiwan and India [16, 32], Iran [30], and Tehran [2]. Other important foodborne bacteria commonly identified in this study were* B. cereus*. Cockroaches' carriage of such kind of bacteria was shown by studies in Botswana [12], Ethiopia [13], Iran [2, 33], and Nigeria [25]. The carriage of* B. cereus* by cockroaches residing in places where food is prepared and stored makes the problem superior as these spore forming bacteria could be a cause of food poisoning outbreak.

*S. aureus* was also resistant against most of the antimicrobial drugs tested with a range of resistance that varies from 20% to 100%. MRSA and VRSA isolates in this study from crawling cockroaches may indicate the ability of cockroaches to acquire strains from the hospital area and crawl back to the community since these antibiotics are mostly applied in the nosocomial setting. Vancomycin is the current choice of antibiotics for resistant* S. aureus* infection and MRSA currently becoming area of concern both in hospital and in community setups.

## 5. Conclusions

*Blattella germanica* were the only cockroaches caught foraging in the FHEs of Jimma town. These cockroaches were identified as carrier of different foodborne bacterial pathogens like* Salmonella* spp.,* S. flexneri*,* E. coli*,* S. aureus*, and* B. cereus*. The guts of cockroaches harbored more foodborne pathogenic bacteria compared to the external body surfaces.

Therefore, environmental sanitation and standard hygiene should be applied in the FHEs. Moreover, health education on the risk inherent in harboring the cockroaches is pertinent to the people working at FHEs.

## Figures and Tables

**Table 1:** Magnitude of cockroach infestation in the FHEs of Jimma town from May 1 to September 30, 2014, Jimma, Southwest Ethiopia.

	Magnitude of cockroach infestation		
	Restaurant	Cafeterias	Total
	Number (%)	Number (%)	Number (%)
*Trapping areas*			
Kitchen	523 (49.9)	224 (28.3)	747 (40.6)
Behind coffee machine	*∗∗*	317 (40)	317 (17.2)
Food preparation area	194 (18.5)	99 (12.5)	293 (15.9)
Refrigerator	45 (4.3)	37 (4.7)	82 (4.5)
Bathroom	122 (11.6)	*∗∗*	122 (6.6)
Toilet	165 (15.7)	115 (14.5)	280 (15.2)

*Total *	*1049 (57)*	*792 (43)*	*1841 (100)*

*∗∗* = not applicable (coffee machines in restaurants and bathrooms in cafeteria were not included in the study).

**Table 2:** Distribution of foodborne bacterial pathogens isolated from *Blattella germanica* cockroaches (*n* = 228 pool of ten) trapped at the FHEs of Jimma town from May 1 to September 30, 2014, Jimma, Southwest Ethiopia.

Isolates	External	Gut	Total
*Salmonella B*	3	6	9
*Salmonella D*	2	3	5
*Salmonella C1*	—	1	1
*Salmonella E*	—	3	3
*Salmonella NG*	1	3	4
*E. coli *	19	16	35
*S. flexneri*	2	1	3
*S. aureus *	5	10	15
*B. cereus*	7	9	16

*Total*	*39*	*52*	*91*

**Table 3:** Medically important bacteria identified from body of cockroaches (*n* = 114 batches) at the FHEs of Jimma town (May 1 to September 30, 2014), Jimma, Southwest Ethiopia.

Isolates	External	Gut	Both^*∗*^	Total
	Number (%)	Number (%)	Number (%)	Number (%)
*Klebsiella *spp.	49 (43)	44 (38.6)	10 (8.8)	93 (40.8)
*Enterobacter *spp.	25 (21.9)	20 (17.5)	13 (11.4)	45 (19.7)
*K. pneumonia*	24 (21.1)	20 (17.5)	6 (5.3)	44 (19.3)
*P. mirabilis*	11 (9.6)	16 (14)	3 (2.6)	27 (11.8)
*P. vulgaris*	8 (7)	14 (12.3)	3 (2.6)	22 (9.6)
*Citrobacter freundii*	8 (7)	15 (13.2)	2 (1.75)	23 (10.1)
*S. marcescens*	9 (7.9)	6 (5.3)	2 (1.8)	15 (6.6)
*Edwardsiella tarda*	10 (8.8)	5 (4.4)	—	15 (6.6)
*Citrobacter diversus*	7 (6.1)	8 (7)	—	15 (6.6)
*P. aeruginosa*	10 (8.8)	—	—	10 (4.4)
*Bacillus *spp.	22 (19.3)	41 (36)	10 (8.8)	63 (27.6)
*Coagulase negative staphylococci*	25 (21.9)	37 (32.5)	7 (6.1)	62 (27.2)
*B. subtilis*	—	9 (7.9)	—	9 (3.9)

*Total *	*208 (47)*	*235 (53)*	*56 (12.6)*	*443 (100)*

Both^**∗**^: the number of similar isolates identified from each one of the surface and gut homogenates of individual batch of cockroaches.
